# Deletion of accessory genes 3a, 3b, 5a or 5b from avian coronavirus infectious bronchitis virus induces an attenuated phenotype both *in vitro* and *in vivo*

**DOI:** 10.1099/jgv.0.001130

**Published:** 2018-08-01

**Authors:** Andrea Laconi, Steven J. van Beurden, Alinda J. Berends, Annika Krämer-Kühl, Christine A. Jansen, Dieuwertje Spekreijse, Gilles Chénard, Hans-Christian Philipp, Egbert Mundt, Peter J. M. Rottier, M. Hélène Verheije

**Affiliations:** ^1^​ Faculty of Veterinary Medicine, Department Pathobiology, Pathology Division, Utrecht University, Utrecht, 3584CL, The Netherlands; ^2^​ Boehringer Ingelheim Veterinary Research Center GmbH & Co. KG, Hannover, Germany; ^3^​ Faculty of Veterinary Medicine, Department Infectious Diseases and Immunology, Immunology Division, Utrecht University, Utrecht, 3584CL, The Netherlands; ^4^​ Boehringer Ingelheim Animal Health Operations BV, Weesp, The Netherlands; ^5^​ Faculty of Veterinary Medicine, Department Infectious Diseases and Immunology, Virology Division, Utrecht University, Utrecht, 3584CL, The Netherlands; ^†^​ Present address: Gupta Strategists, Ophemert, The Netherlands.; ^‡^​ Present address: Intravacc Animal Research Centre, Ponwalla Science Park, The Netherlands.; ^§^​ Present address: Thermo Fisher Scientific, 8211AR Lelystad, The Netherlands.

**Keywords:** infectious bronchitis virus, Coronavirus, chicken, accessory genes, accessory proteins, live attenuated virus

## Abstract

Avian coronavirus infectious bronchitis virus (IBV) infects domestic fowl, resulting in respiratory disease and causing serious losses in unprotected birds. Its control is mainly achieved by using live attenuated vaccines. Here we explored the possibilities for rationally attenuating IBV to improve our knowledge regarding the function of IBV accessory proteins and for the development of next-generation vaccines with the recently established reverse genetic system for IBV H52 based on targeted RNA recombination and selection of recombinant viruses in embryonated eggs. To this aim, we selectively removed accessory genes 3a, 3b, 5a and 5b individually, and rescued the resulting recombinant (r) rIBV-Δ3a, rIBV-Δ3b, rIBV-Δ5a and rIBV-Δ5b. *In vitro* inoculation of chicken embryo kidney cells with recombinant and wild-type viruses demonstrated that the accessory protein 5b is involved in the delayed activation of the interferon response of the host after IBV infection. Embryo mortality after the inoculation of 8-day-old embryonated chicken eggs with recombinant and wild-type viruses showed that rIBV-Δ3b, rIBV-Δ5a and rIBV-Δ5b had an attenuated phenotype *in ovo*, with reduced titres at 6 h p.i. and 12 h p.i. for all viruses, while growing to the same titre as wild-type rIBV at 48 h p.i. When administered to 1-day-old chickens, rIBV-Δ3a, rIBV-Δ3b, rIBV-Δ5a and rIBV-Δ5b showed reduced ciliostasis in comparison to the wild-type viruses. In conclusion, individual deletion of accessory genes in IBV H52 resulted in mutant viruses with an attenuated phenotype.

## Introduction

Infectious bronchitis virus (IBV) is primarily a respiratory pathogen of domestic fowl and is the cause of economic losses worldwide. It enters the host through the respiratory tract, causing the destruction of the epithelium with consequent respiratory distress and inclination towards the development of secondary bacterial infections [1]. Depending on the genotype, IBV can cause other clinical signs, including severe nephritis, ‘false layer’ syndrome and proventriculitis [1].

IBV is a member of the genus *Gammacoronavirus*, family *Coronaviridae*, order *Nidovirales*. It is an enveloped virus containing a positive-sense RNA genome that is 27.6 kb in length, coding for at least 10 open reading frames (ORFs) characterized by the following organization: 5′UTR-1a-1ab-S-3a-3b-E-M-5a-5b-N-3′UTR. The first two-thirds of the genome comprises the replicase gene, which is expressed through 2 polyproteins, pp1a and pp1ab, which are cleaved by 2 types of virus-encoded proteinases, resulting in 15 non-structural proteins. In addition, the genome encodes four structural proteins, spike glycoprotein (S), small membrane protein (E), integral membrane protein (M) and nucleocapsid protein (N), and a set of accessory proteins [2].

The presence of accessory proteins is a common feature of the members of the family *Coronaviridae*, although differences in number, location along the genome and amino acid composition have been observed between viruses belonging to this family [3]. With the advent of reverse genetics systems (RGS) for coronaviruses, accessory proteins have been proved to be generally dispensable for replication *in vitro* [4–8]. Furthermore, some of these studies have shown that coronavirus accessory proteins are involved in virus–host interactions during coronavirus infection *in vivo* [5, 6, 8]. Until recently such studies were not possible for IBV, as the only reverse genetics system available was based on Beaudette, a non-pathogenic and highly cell-adapted strain that is unable to replicate *in vivo* [9]. In our laboratories a RGS for IBV strain H52 has been recently developed, based on targeted RNA recombination and the rescue and selection of recombinants in embryonated chicken eggs [10]. The rIBV H52 lacking the gene clusters 3ab and/or 5ab [11], showed a remarkable reduction in virulence in 1-day-old chicks. In addition, they had the ability to induce sufficient immune responses to protect against virulent IBV of the Mass genotype upon challenge [11]. However, the deletion of complete gene clusters did not allow us to investigate the involvement of each individual accessory protein in the reduction of virulence.

Here, we selectively deleted individual accessory genes of IBV H52 in order to assess how the absence of a single protein influences the phenotype of the virus *in vitro*, *in ovo* and *in vivo*. The data gathered provide useful information regarding the significance of IBV’s accessory proteins, and increase the options towards the development of a new generation of rationally attenuated live vaccines.

## Results

### Generation of rIBV-Δ3a, rIBV-Δ3b, rIBV-Δ5a and rIBV-Δ5b

**Fig. 1.**
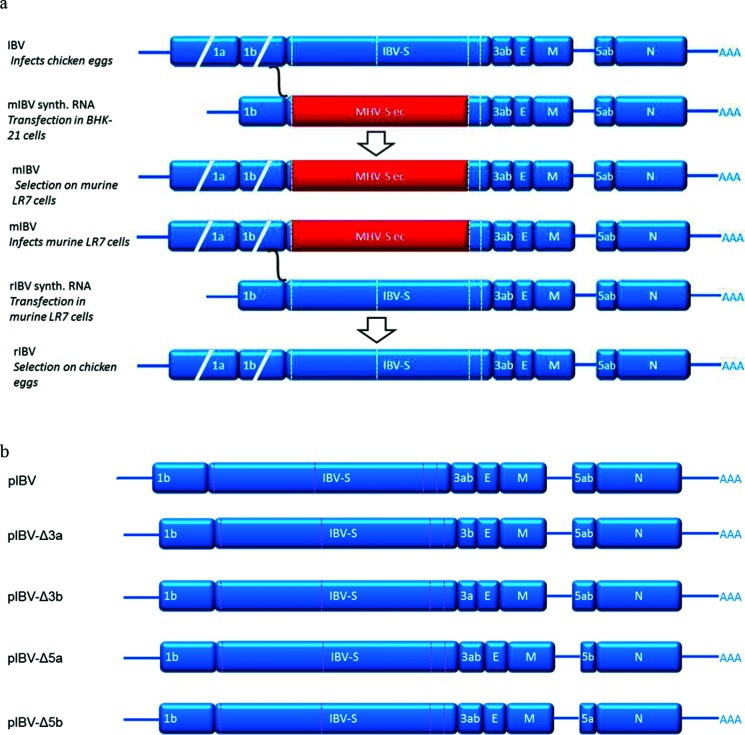
Schematic overview of the targeted RNA recombination principle and the donor plasmids used in the present study. (a) Schematic overview of the targeted RNA recombination method to generate recombinant rIBV-wt [10]. IBV sequences are in blue and murine hepatitis virus (MHV) sequences are in red. (b) Schematic layout of the donor plasmids pIBV-Δ3a, pIBV-Δ3b, pIBV-Δ5a or pIBV-Δ5b used in targeted RNA recombination to generate rIBV-Δ3a, rIBV-Δ3b, rIBV-Δ5a and rIBV-Δ5b, respectively.

**Fig. 2.**
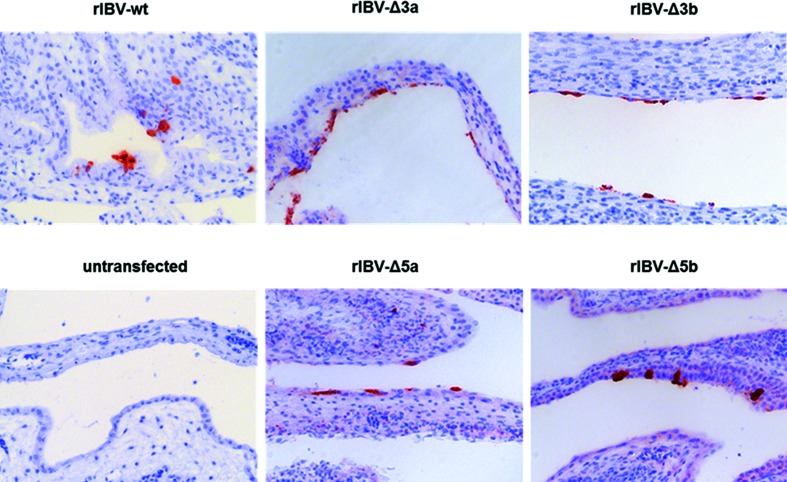
Immunohistochemistry of CAMs after rescue of rIBV-Δ3a, rIBV-Δ3b, rIBV-Δ5a and rIBV-Δ5b. Embryonated chicken eggs were inoculated with mIBV-infected LR7 cells that had been transfected with *in vitro* transcripts from donor plasmids of pIBV-Δ3a, pIBV-Δ3b, pIBV-Δ5a or pIBV-Δ5b by electroporation. mIBV-infected non-transfected LR7 cells served as controls. Formalin-fixed and paraffin-embedded CAMs were stained using a monoclonal antibody against IBV-S2.

Eight-day-old embryonated chicken eggs (ECEs) were inoculated with LR7 cells that had been infected with murine IBV (mIBV) and transfected with *in vitro-*transcribed donor RNA obtained from p-IBV-Δ3a, p-IBV-Δ3b, p-IBV-Δ5a and p-IBV-Δ 5b, respectively. At 7 days post-inoculation embryonic death was observed in all the groups. The embryos showed stunting and curling typical for IBV infection. Replication of rIBV-Δ3a, rIBV-Δ3b, rIBV-Δ5a and rIBV-Δ5b (schematically depicted in Fig. 1) was confirmed by RT-qPCR on viral RNA extracted from the allantoic fluid (AF) (data not shown). Virus replication in chorio-allantoic membrane (CAMs) from eggs inoculated with transfected cells was demonstrated by the detection of viral antigens by IHC (Fig. 2), while eggs that had received non-electroporated control cells showed no viral antigen production (Fig. 2).

### Genetic identity and stability rIBV-Δ3a, rIBV-Δ3b, rIBV-Δ5a and rIBV-Δ5b

**Fig. 3.**
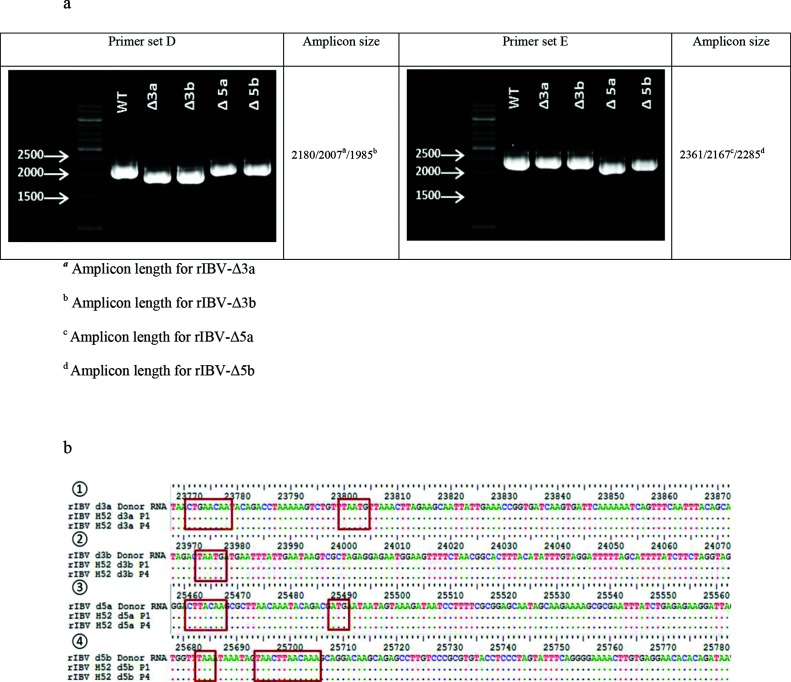
Genetic characterization and stability of rescued recombinant virus rIBV-Δ3a, rIBV-Δ3b, rIBV-Δ5a and rIBV-Δ5b. (a) Electrophoresis showing the amplicons obtained after the amplification of cDNA templates of viral RNA extracted from the AF of ECEs inoculated with P1 of virus rIBV-Δ3a, rIBV-Δ3b, rIBV-Δ5a and rIBV-Δ5b using primer sets that span the genomic area of accessory genes 3 (primer set D) and 5 (primer set E). Expected amplicon sizes are indicated in the right columns. (b) Sequences and locations of the genomic regions in which the mutations were introduced in order to selectively delete the accessory genes. (1) Comparison of the sequences obtained from P1 and P4 of rIBV-Δ3a. The genomic region shown is the junction between the spike gene and the 3b gene; the TRS (CTGAACAA) now used for the transcription of genes 3b and E and the overlap between the S gene stop codon and the 3b gene codon start (TAATG), respectively, are highlighted in the red squares. (2) Comparison of the sequences obtained from P1 and P4 of rIBV-Δ3b. The genomic region shown is the junction between the 3a and the E genes; the overlap between the 3a gene stop codon and the E gene start codon (TAATG) is highlighted in the red square. (3) Comparison of the sequences obtained from P1 and P4 of rIBV-Δ5a. The genomic region shown is the junction between the intergenic region and the 5b gene; the TRS (CTTAACAA) and the start codon (ATG) now used for the transcription of gene 5b, respectively, are highlighted in the red squares. (4) Comparison of the sequences obtained from P1 and P4 of rIBV-Δ5b. The genomic region shown is the junction between the 5a and the 5b genes; the two stops codon (TAA) introduced to prevent the transcription of gene 5b and the TRS (CTTAACAA) used for the transcription of gene N, respectively, are highlighted in the red squares.

The genetic identity of the viruses was evaluated by RT-PCR targeting the genomic regions where the mutations were introduced. Sequencing of amplicons obtained with primer set D (Table 1) confirmed the deletion of genes 3a and 3b, while primer set E (Table 1) confirmed the deletion of gene 5a and the presence of the mutations introduced in the 5b gene sequence to suppress the expression of the encoded protein (Fig. 3). Viruses present in the allantoic fluid of infected eggs matching the expected sequence in the mutated region of the genome were selected and passaged three additional times in ECEs to generate virus working seeds and stocks. Sequence analysis of the 3′ 9 kb of working stock passage 4 of all recombinant viruses confirmed the expected sequence, with the exception of rIBV-Δ3b, where a mutation (A→T) occurred in the 3′ UTR (position 27 160), and of rIBV- Δ5a, where a mutation (A→T) within the 1ab gene (position 19794) caused a coding change (Asn→Tyr).

**Table 1.** Primer sets used for the characterization of the 3′ 9 kb of the viral genome of rIBVs

Primer set	Primer	Sequence (5′ → 3′)	Amplicon (bp)*^a^*
A	IBV.F12	TGTCAAGATGTCAACTGG	2327
	IBV.R12	GCATTCACTGCTGTACAG	
B	IBV.F13	ACAGAGCACAAGTTTGATC	1721
	IBV.R13	CGCTCTTAGTAACATAAAC	
C	IBV.F14	TAAATGGTGATCTTGTTT	2299
	IBV.R14	AACACTATACCATTAGGTGC	
D	IBV.F15	TGCTGCTTCCTTTAATAAG	2180/2007*^a^*/1985*^b^*
	IBV.R37	GAGAAAGCACCATTGGCACA	
E	IBV.F16	CTTAACATTGCAGTAGGTG	2361/2167*^c^*/2285*^d^*
	IBV.R16	CTGAGGTCAATGCCTTATC	
F	IBV.F28	TGTTGTAGGTTGTGGTCCCA	2171
	IBV.R31	CTAATGGGCGTCCTAGTGCT	

*a,* amplicon length for rIBV-Δ3a.

*b,* amplicon length for rIBV-Δ3b.

*c,* amplicon length for rIBV-Δ5a.

*d,* amplicon length for rIBV-Δ5b.

### Recombinant IBVs have comparable growth kinetics in embryonated eggs

**Fig. 4.**
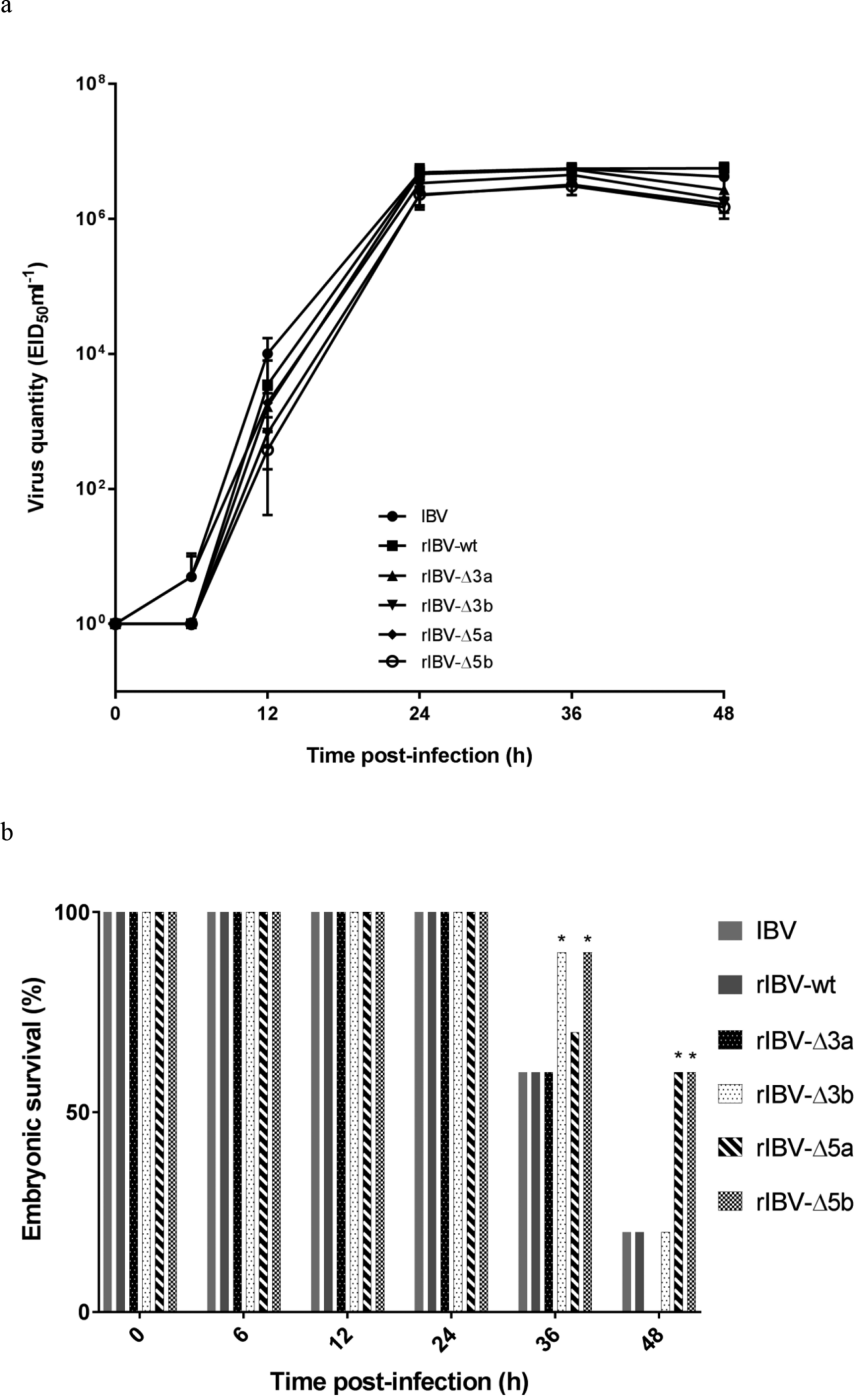
*In ovo* characteristics of rIBVs. (a) Quantitative RT-qPCR analysis was performed on RNA extracted from the AF of ECEs collected at 12 h intervals after inoculation with IBV H52 BI, rIBV-wt rIBV-wt, rIBV-Δ3a, rIBV-Δ3b, rIBV-Δ5a and rIBV-Δ5b. The data points represent means with standard deviations of five eggs per condition, with all samples being run and analysed in triplicate using a 10-fold dilution series of IBV H52 BI as a reference to determine virus quantity as EID_50_ ml^−1^ equivalents. (b) Embryonic death is indicated as a percentage of all remaining animals at each time point. *, significant difference (*P*<0.05) in comparison with wild-type H52 viruses.

The *in ovo* growth kinetics of the recombinant viruses were assessed by inoculating 8-day-old ECEs with 10^2^ EID_50_ of working stock per egg and determining the relative viral load in the AF of five eggs per virus at 6, 12, 24, 36 and 48 h post-infection (p.i.) by RT-qPCR. At the time points 6 and 12 h p.i. the viral loads observed for all viruses in which one of the accessory genes was deleted were slightly lower than for IBV H52 BI and rIBV-wt (Fig. 4a), while at a later stage (24–48 h p.i.), the viral loads were comparable for all of the viruses tested, indicating that the viruses propagated to comparable titres.

### rIBV-Δ3b, rIBV-Δ5a and rIBV-Δ5b show an attenuated phenotype in embryonated eggs

The embryo mortality for 8-day-old ECEs was evaluated at 6, 12, 24, 36 and 48 h p.i. with 10^2^ EID_50_ of IBV H52 BI, rIBV-wt, rIBV-Δ3a, rIBV-Δ3b, rIBV-Δ5a, or rIBV-Δ5b. No embryonic death was observed within the first 24 h p.i. for any virus. At 36 h p.i. 60 % of the eggs infected with wild-type viruses and rIBV-Δ3a were alive, while the percentage of vital embryos was 70 % for rIBV-Δ5a and 90 % for rIBV-Δ3b and rIBV-Δ5b. At 48 h p.i. embryo vitality was 20 % or less for wild-type viruses and rIBV-Δ3b, 60 % for both rIBV-Δ5a and rIBV-Δ5b, and no embryos were alive at 48 h p.i. with rIBV-Δ3a (Fig. 4b).

### rIBV-Δ5b induces an early type I interferon response

**Fig. 5.**
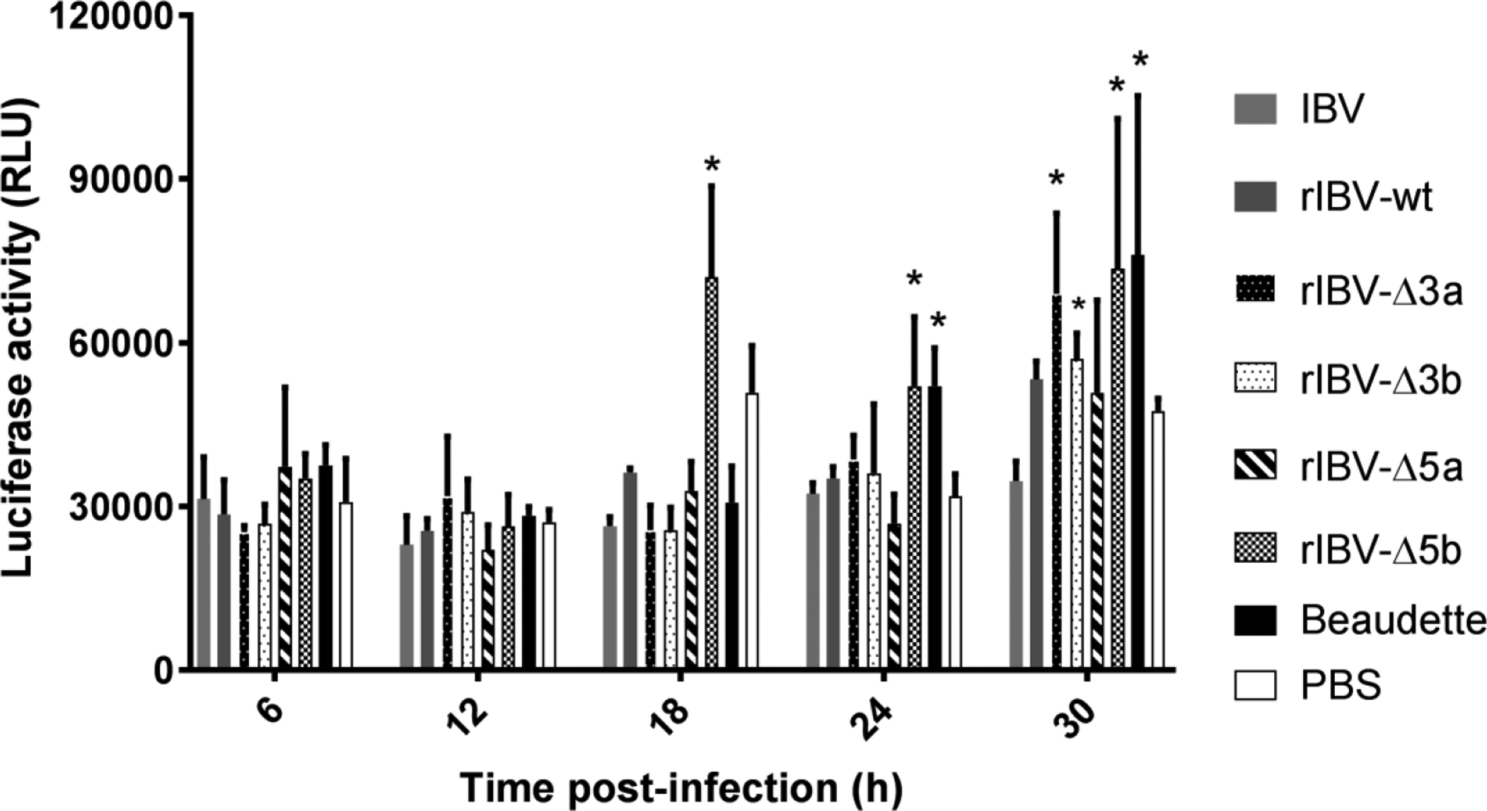
CEK cells were infected with Beaudette, IBV H52 BI, rIBV-wt, rIBV-Δ3a, rIBV-Δ3b, rIBV-Δ5a and rIBV-Δ5b at a multiplicity of infection (m.o.i.) of 10 in triplicate. Culture supernatants were collected at five time points and used to infect CEC-32 cells in duplicates. The values represent the means of six measurements for each virus at each time point, and the bars indicate sd. *, significant differences (*P*<0.05) as compared to wild-type H52 viruses.

The type I interferon response was evaluated for each virus at 6, 12, 18, 24 and 30 h after the inoculation of chicken embryonic kidney cells (CEKs). The supernatants were collected from CEKs at the different time points and used to inoculate cell culture wells containing CEC-32 cells, a reporter cell line expressing luciferase under the control of an IFN-responsive chicken Mx promoter. A one-way analysis of variance (ANOVA) with Tukey’s honestly significant difference (HSD) post hoc test was used to assess whether the differences between the deletion mutants and the wild-type viruses were significant. The results are reported in Fig. 5. No significant differences between the different viruses were observed at the first two time points. However, rIBV-Δ5b showed an early onset (18 h p.i.) of type I IFN production (*P*<0.05), as indicated by luciferase activity, in comparison not only to the wild-type viruses, but also to the other deletion mutants and IBV Beaudette. The luciferase activity measurements following IBV-Δ5b infection were also the highest of those for the recombinant viruses at the two following time points. A significant difference in the luciferase activity between rIBV-Δ3a or rIBV-Δ3b and the wild-type viruses was only observed at 30 h p.i., while rIBV-Δ5a showed similar luciferase activity to the wild-type viruses at each time point.

### IBV deletion mutants’ phenotype *in vivo*

**Fig. 6.**
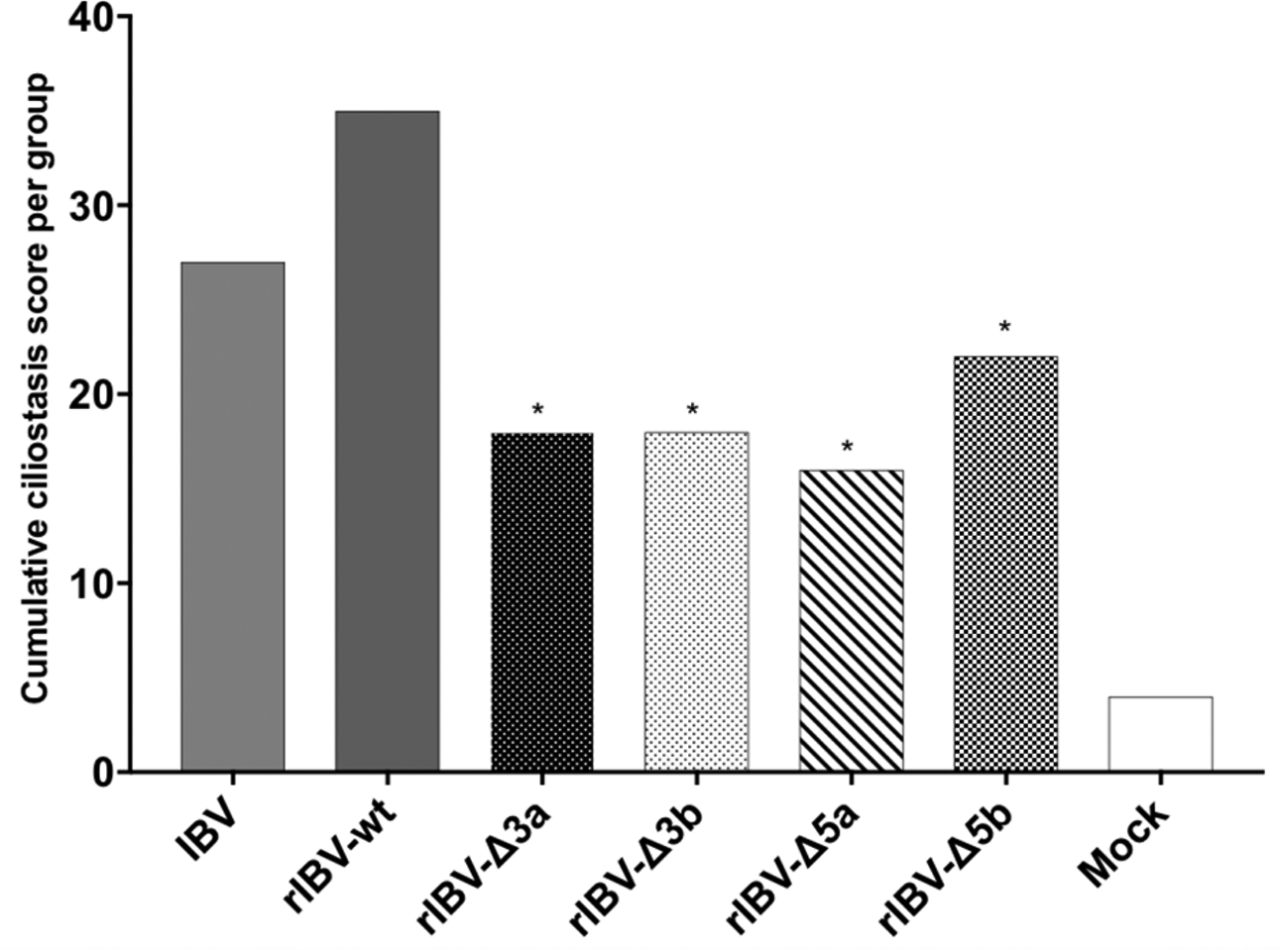
Ciliostasis after vaccination with IBV H52 BI and rIBVs in chickens. The maximal ciliostasis score per animal is 40, which indicates complete ciliostasis in all 10 transversal tracheal sections examined. Ciliostasis in 1-day-old chickens determined 7 days after vaccination with IBV H52 BI, rIBV-wt, rIBV-Δ3a, rIBV-Δ3b, rIBV-Δ5a, rIBV-Δ5b, or non-vaccinated controls. *, significant difference (*P*<0.05) in comparison with wild-type H52 viruses.

The phenotype of the deletion mutants was assessed *in vivo* by inoculation in 1-day-old SPF chickens with a dose of 10^3^ EID_50_ per animal of IBV H52 BI, rIBV-wt, rIBV-Δ3a, rIBV-Δ3b, rIBV-Δ5a, or rIBV-Δ5b. As a readout for attenuation, the ciliary activity of 10 tracheal sections per bird at 7 days post-inoculation was evaluated. All rIBV deletion mutants showed reduced ciliostasis compared to wild-type H52 and recombinant H52, with rIBV-Δ5a showing the most attenuated phenotype (Fig. 6). Statistical analysis showed a significant difference (*P*<0.05) in ciliary activity between the deletion mutants and rIBV-wt in infected chickens (Fig. 6). There was no significant difference between the two wild-type viruses. The cumulative ciliostasis scores from rIBV-Δ3a-, rIBV-Δ3b-, rIBV-Δ5a-, or rIBV-Δ5b-vaccinated animals differed from those of the non-vaccinated controls (*P*<0.05).

## Discussion

In the present study, the recently developed RGS (based on targeted RNA recombination and selection in embryonated chicken eggs) of IBV H52 BI was used either to remove the accessory genes encoding the 3a, 3b and 5a proteins or to inactivate 5b protein expression. We performed a comprehensive analysis that showed for the first time that the four accessory proteins of IBV all contribute to the pathogenicity of the virus.

To this aim, the single-deletion mutants were inoculated in 8-day-old embryonated chicken eggs, in which they showed a slightly delayed but otherwise similar growth kinetics compared to those of the wild-type viruses, hence confirming that IBV accessory proteins are not essential for viral replication in embryonated chicken eggs [5, 8, 11, 12]. Differences in embryo mortality were observed, with rIBV-Δ3b, rIBV-Δ5a and rIBV-Δ5b showing an attenuated phenotype in comparison to the wild-type viruses (IBV H52 BI and rIBV-wt).

Subsequently we investigated whether IBV accessory proteins are involved in evading the immune response of the host. Viral replication in a host relies on the ability of the virus to delay or counteract the type I IFN response [13] and accessory proteins of other members of the family *Coronaviridae* are known to be involved in the mechanisms to escape the type I IFN response [7, 9, 14, 15]. As the immune system in ECEs becomes fully functional during the last week of embryonic development (from about 14 days of age) [16], we initially tried to assess the type I IFN response in our bioassay using the allantoic fluid collected from 16-day-old ECEs infected with deletion mutants and wild-type viruses. However, the results obtained were inconclusive (data not shown), probably due to the presence of luciferase inhibitors in the AF of the ECEs [17, 18]. We consequently adopted an *in vitro* approach in which we infected CEK cells with rIBVs and used the collected inactivated supernatants from these to perform the type I IFN luciferase bioassay. rIBV-Δ5b showed an earlier onset of type I IFN production in comparison with both the wild-type viruses and the other single-deletion mutants, as indicated by an increase in the luciferase activity. This finding is in accordance with a previous study, which identified protein 5b as being responsible for the shut-off of the host translation machinery for the cell culture-adapted IBV Beaudette [19]. As a consequence of the inhibition of protein translation, no type I IFNs are synthesized. Compared to the wild-type viruses, an early onset in type I IFN response was also observed for rIBV-Δ3a and rIBV-Δ3b (30 h p.i.); protein 3a was previously reported to be involved in counteracting the antiviral IFN response [20], while protein 3b was found to be involved in the inhibition of *Ifnβ* transcription [21]. Taken as a whole, these data are consistent with previous observations in studies using the non-pathogenic strain Beaudette, in which IBV accessory proteins 3a, 3b and 5b acted as type I IFN antagonists.

Subsequently, we assessed whether the inability of the deletion mutants to delay or counteract the type I IFN response *in vitro* corresponded with an attenuated phenotype *in vivo*. We infected 1-day-old SPF chickens with the single mutants and the wild-type viruses and evaluated the ciliary activity at 7 d p.i. All rIBV single mutants showed an attenuated phenotype in comparison to the wild-type viruses. Of all the recombinant viruses tested, rIBV-Δ5a showed the most attenuated phenotype *in vivo*. To the best of our knowledge, this is the first report demonstrating the involvement of protein 5a in the pathogenicity of IBV. The data gathered in this study suggest that protein 5a is not a type I IFN antagonist and that it is dispensable for replication *in ovo*. Similarly, the deletion of gene 6 from SARS-CoV does not affect the replication *in vitro*, but results in lower titres and lower morbidity and mortality *in vivo* [22]. We can speculate that protein 5a acts as a co-factor in the replication *in vivo* and that its deletion results in lower viral replication rates in chickens and consequently in an attenuated phenotype.

The data presented in this study show that each individual IBV accessory protein is accountable for the pathogenicity of the virus, despite being involved in different pathways, and that the deletion of one of these genes is sufficient to cause an attenuated phenotype *in vivo*. Furthermore, we demonstrated that the reverse genetics system for IBV based on targeted RNA recombination allows the rational modification of IBV strains, and that this system could not only be used effectively for studies on gene functions, but also for the generation of rationally attenuated viruses, as the rescued recombinant viruses proved to be less virulent than the wild-type viruses.

## Methods

### Cells, eggs and viruses

Murine LR7 cells [23] were cultured in Dulbecco’s modified Eagle’s medium (DMEM; BioWhittaker) supplemented with 4 mM l-glutamine (Lonza), 10 % foetal calf serum (FCS; BioWhittaker) and 0.05 mg ml^−1^ gentamicin (Gibco Invitrogen) at 37.0 °C and 5 % CO_2_. CEK cells were obtained by aseptically removing the kidneys from 17- or 18-day-old chicken SPF white leghorn egg embryos (Animal Health Service, Deventer, The Netherlands). A cell suspension was obtained by trypsinization and the resulting CEK cells were seeded in 24-well plates at 1.5*10^5^ CEK cell/well in Alpha MEM (Gibco Invitrogen) supplemented with 10 % FCS (BioWhittaker), 4 mM l-glutamine (Lonza) and 0.1 % penicillin/streptomycin (Gibco Invitrogen). The CEC-32 quail reporter cell line expressing luciferase under the control of the chicken *Mx* promoter [24] was kindly provided by Dr S. Härtle, LMU Munich. CEC-32 cells were cultured in Iscove’s modified Dulbecco’s medium (Sigma), 8 % FCS (BioWhittaker), 2 % chicken serum (Gibco Invitrogen) and 1 % penicillin/streptomycin (Gibco Invitrogen) at 37.0 °C and 5 % CO_2_. Specific pathogen-free (SPF) white leghorn eggs (Animal Health Service, Deventer, The Netherlands) were incubated at 37.5 °C and 45–65 % relative humidity. At day 8 of incubation the embryonated chicken eggs (ECE) were inoculated with virus into the allantoic cavity and candled daily. ECEs with dead embryos were transferred to 4 °C for 16–24 h, and then the AF and CAMs were collected. The virus titre *in ovo* was expressed as the 50 % embryonic infectious dose (EID_50_) ml^−1^, as determined at day 7 p.i. according to Reed and Muench [25]. For preparing virus stocks, the AF of 4–10 ECEs inoculated with 100 EID_50_ were pooled after incubation for 24 h.

IBV strain H52 BI (Boehringer Ingelheim, Ingelheim, Germany), recombinant IBV wild-type (rIBV-wt), IBV strain Beaudette (Animal Health Service, Deventer, The Netherlands) and murinized (m)IBV (strain #1B3-IIA) were propagated as described previously [10].

### Immunohistochemistry

Immunohistochemistry (IHC) was performed on CAMs collected from ECEs using monoclonal antibody (MAb) Ch/IBV 26.1 directed against the IBV S2 protein (Prionics, Lelystad, The Netherlands) [10].

### RNA isolation, reverse transcription and PCR

RNA from the AFs of ECEs was isolated using the QIAamp viral RNA Mini kit (Qiagen, Hilden, Germany) according to the manufacturer’s protocol. To assess the relative viral load in the AFs, a semi-quantitative one-step RT-qPCR was performed using a previously described protocol [10]. For sequencing and cloning purposes, reverse transcription was performed using the Transcriptor First Strand cDNA Synthesis kit (Roche, Basel, Switzerland) according to the manufacturer’s protocol, while PCR was performed with Phusion Hot Start II High-Fidelity DNA polymerase (Thermo Fisher Scientific).

### Construction of p-IBV-Δ3a, p-IBV-Δ3b, p-IBV-Δ5a and p-IBV-Δ5b

The donor plasmid was designed as previously described [10]. The constructs p-IBV-Δ3a, p-IBV-Δ3b, p-IBV-Δ5a and p-IBV-Δ5b, in which the accessory genes 3a, 3b, 5a, or 5b were removed, and the wild-type plasmid are shown in Fig. 1. The plasmids used for the generation of the donor plasmids p-IBV-Δ3a, p-IBV-Δ3b, p-IBV-Δ5a and p-IBV-Δ 5b are listed in Table 2. The delta3a fragment (Δ3a) was designed such that the 1 nt overlap between the stop codon of the spike gene and the start codon of the 3a gene was replaced by an overlap between the stop codon of the spike gene and the start codon of the 3b gene. The design of the delta3b fragment (Δ3b) was such that the 1 nt overlap between the stop codon of the 3b gene and the start codon of the E gene was replaced by an overlap between the stop codon of the 3a gene and the start codon of the E gene. The delta5a fragment (Δ5a) was designed such that the start codon of the 5a gene is now used as start codon of the 5b gene. Finally, the delta5b fragment (Δ5b) was designed such that the 5b start codon was silently removed and an extra stop codon was introduced. The transcription regulatory sequence (TRS) used by the N gene is located within the 5b gene, therefore this part of the gene sequence was not further deleted or modified. DNA fragments designed as described above were cloned into pUC57-simple by Genscript (Piscataway, NJ, USA) and underwent a series of digestion ligation steps to obtain the full donor plasmids, as described previously [10]. After each ligation step the inserted region was amplified by PCR and sequenced (Macrogen, Amsterdam, The Netherlands).

**Table 2.** Plasmids used for generation of the donor plasmids p-IBV-Δ3a, p-IBV-Δ3b, p-IBV-Δ5a and p-IBV-Δ 5b

Plasmid	Genes	Coordinates	Length (nt)	3′-end RES	Surrounding RES	Inserted in plasmid
p-IBV-5	5′-UTR	1…497	497	*Bst*BI	n.a.	n.a.
p-IBV-1b	1b, S	19 610…20 379	770	*Xho*I	*Bst*BI	p-IBV-5
p-IBV-S	S	20 379…23 590	3211	*Sty*I	*Xho*I	p-IBV-5-1b
p-IBV-SIR	S, 3ab, E, M	23 591…25 318	1728	*Eco*RI	*Sty*I	p-IBV-5-1b-S
p-IBV-3T	5ab, N, 3′-UTR	25 319…27 730	2422	*Mss*I, *Pac*I	*Eco*RI	p-IBV-5-1b-S-SIR
p-Δ3a	n.a.	23 604…24 304	528	n.a.	*Nhe*I, *Pml*I	p-IBV-5-1b-S-SIR
p-Δ3b	n.a.	23 604…24 304	524	n.a.	*Nhe*I, *Pml*I	p-IBV-5-1b-S-SIR
p-Δ5a	n.a.	25 467…25 974	314	n.a.	*Afe*I, *Nhe*I	p-IBV-3T
p-Δ5b	n.a.	25 467…25 974	433	n.a.	*Afe*I, *Nhe*I	p-IBV-3T

### Targeted RNA recombination and rescue of recombinant IBVs

As described previously [10], capped run-off donor transcripts were synthesized from the *Mss*I-linearized plasmids p-IBV-Δ3a, p-IBV-Δ3b, p-IBV-Δ5a and p-IBV-Δ5b using the mMessage mMachine T7 kit (Ambion by Thermo Fisher Scientific). The *in vitro*-transcribed RNA was transfected by electroporation into LR7 cells previously infected with mIBV, allowing the introduction of the IBV spike ectodomain into mIBV genome by targeted RNA recombination, as previously described [10]. IBV recombinant viruses were then selected via intra-allantoic injection in 8-day-old ECEs using five eggs per dilution (10^−1^–10^−5^) and replication was assessed by IHC and RT-qPCR. After sequence analysis of the modified regions, recombinant viruses underwent two further rounds of end-point dilution in 8-day-old ECEs, and virus stocks were grown and titrated. The entire 3′ end (from nt 18 612 onwards) of passage 4 of all rIBV delta variants was sequenced using the oligo sets reported in Table 1.

### *In ovo* growth kinetics and phenotype of rIBV-Δ3a, rIBV-Δ3b, rIBV-Δ5a, and rIBV-Δ5b

Comparison of the growth kinetics of rIBV-Δ3a, rIBV-Δ3b, rIBV-Δ5a, and rIBV-Δ5b against those of H52 BI and rIBV-wt was performed by RT-qPCR on viral RNA extracted from the AF of inoculated 8-day-old ECEs. Previously a standard curve was obtained using a 10-fold dilution series of IBV H52 BI with known EID_50_ ml^−1^ [10]. Embryo death was evaluated at each time point in order to assess the phenotype of single-deletion mutants. A one-way ANOVA with Tukey’s HSD post hoc test was performed to analyse whether embryonic death was statistically different between the groups.

### Infection of CEK cells with rIBV-Δ3a, rIBV-Δ3b, rIBV-Δ5a and rIBV-Δ5b

CEK cells obtained from kidneys of 16-day-old ECEs were seeded in 24-well plates and inoculated at an m.o.i. of 10 with the following viruses: IBV Beaudette, IBV H52 BI, rIBV-wt, rIBV-Δ3a, rIBV-Δ3b, rIBV-Δ5a and rIBV-Δ5b. Supernatant from infected cells was collected at 6-h intervals starting at 6 h p.i. Cells to which PBS of the same volume as the AF was added served as negative controls.

### Chicken type I interferon bioassay

The chicken type I interferon (chIFN) response was assessed using a bioassay based on the CEC-32 quail reporter cell line expressing luciferase under the control of the chicken *Mx* promoter [24]. First, the samples were heat-inactivated (56 °C for 30 min) in order to avoid the influence of virus on the assay. CEC-32 cells were seeded at 1×10^5^ cells/well and incubated with the supernatant of CEK-infected cells for 6 h. Luciferase activity was measured using a luciferase assay kit (Promega) following the manufacturer’s instructions. A standard curve was created by culturing CEC-32 with a twofold dilution series of a known concentration of recombinant IFNα (6 U ml^−1^). A one-way ANOVA with Tukey's HSD post hoc test was performed to analyse whether the luciferase activities were statistically different between the groups. A *P* value <0.05 was considered statistically significant.

### Assessment of *in vivo* phenotype of single delta variants

One hundred layer-type chickens were hatched from SPF white leghorn eggs at BIAHO from eggs obtained from Valo Biomedia, Osterholz-Scharmbeck, Germany. At 1 day of age the birds were divided into seven groups and kept in separate isolators under controlled housing conditions. Animals were inoculated via eye-drop with 10^3^ EID_50_ in 0.1 ml of IBV H52 BI (*n*=15), rIBV-wt (*n*=15), rIBV-Δ3a (*n*=15), rIBV-Δ3b (*n*=15), rIBV-Δ5a (*n*=15) and rIBV-Δ5b (*n*=15), or not inoculated (*n*=10, negative control), respectively. At 7 days post-infection the animals were humanely killed for sampling of the trachea and subsequent evaluation of the ciliary activity. Briefly, for each trachea ten 1 mm transverse sections were collected (three upper, four middle and three lower) and examined under low-power microscopy. The ciliostasis of each tracheal section was scored on a scale from 0 (100 % ciliary activity) to 4 (no ciliary activity), and the average ciliostasis score was calculated for each group of animals [26]. Eventually a one-way ANOVA with Tukey's HSD post hoc test was performed to analyse whether the average ciliostasis scores between the groups were statistically different. A *P* value <0.05 was considered statistically significant
